# Fabricate of High-Strength and High-Conductivity Cu–Cr–Si Alloys through ECAP-Bc and Aging Heat Treatment

**DOI:** 10.3390/ma13071603

**Published:** 2020-04-01

**Authors:** Tingbiao Guo, Junjie Wang, Yibo Wu, Xiaoyang Tai, Zhi Jia, Yutian Ding

**Affiliations:** 1 State Key Laboratory of Advanced Processing and Recycling of Nonferrous Metals, Lanzhou University of Technology, Lanzhou 730050, Gansu, China; wjj960831@outlook.com (J.W.); 15027748144@163.com (Y.W.); txy052352@outlook.com (X.T.); jiazhi@lut.edu.cn (Z.J.); dingyt@lut.edu.cn (Y.D.); 2 School of Materials Science and Engineering, Lanzhou University of Technology, Lanzhou 730050, Gansu, China

**Keywords:** Cu–1Cr–0.2Si alloy, equal channel angular pressing (ECAP), electron back-scattered diffraction (EBSD), texture, mechanical properties, electrical conductivity

## Abstract

The effect of equal channel angular pressing (ECAP) through the route Bc and aging treatment on the grain structure and properties of the Cu–1Cr–0.2Si alloy was investigated. Microstructure was detected by scanning electron microscopy (SEM), x-ray diffraction (XRD), and electron backscatter diffraction (EBSD) and the mechanical properties and electrical conductivity were tested. Results shown that after ECAP, accompanying the grains refined to nano-and submicron-structure, the Cr particles were gradually spread along the grain boundaries (GBs), aging treatment promoted Cr particles dispersed in the matrix. ECAP greatly increased the ultimate tensile strength (UTS) while having a small effect on the conductivity, and aging treatment increased electrical conductivity. The stable {111}<110> texture after ECAP and the lower dislocation density after aging treatment maybe the main reasons for the high conductivity of the material.

## 1. Introduction

Cu–Cr alloys are widely used in integrated circuit lead frames, electric locomotive contact lines, and other fields due to its excellent electrical, thermal conductivity, and high strength [1,2,3]. The mechanical properties of the alloy can be improved by precipitation strengthening, however, the Cr element has a low solid solubility in Cu, especially at room temperature or lower Cr alloys [4], which makes it difficult to increase the strength of the as-cast Cu–Cr alloys. Therefore, multi-element composite strengthening has gradually attracted much attention. Tian et al. [5] found that the Zr element can inhibited the formation of the eutectic Cr phase, which refines the dendritic Cr phase and generates flaky Cu–Zr intermetallic compounds that significantly improve the thermal stability of the material. Batawi et al. [6] found that the addition of Mg and Zr can change the order of precipitation phase in Cu–Cr alloys and increase the materials’ peak strength. Si has an effect on the deoxidation purification and solid solution strengthening of Cu–Cr alloys. The Ni_2_Si phase, which forms after the addition of Si and Ni elements, can further refine the precipitated phase particles and effectively increase the tensile strength and softening resistance of the alloy, but has little effect on the electrical conductivity [7,8].

Severe plastic deformation (SPD) can effectively improve the microstructure of metal materials such as Al alloys, Cu alloys, Mg alloys, etc., and greatly improve the uniformity of microstructure [9], however, ECAP can not only improve the uniformity of microstructure, but can also greatly improve the tensile strength without affecting the conductivity [10,11,12]. Annealing treatment can promote the formation of the strengthening phase distributing in the matrix, which is effective at improving the electrical conductivity [13,14]. Wei et al. [15] obtained a Cu–0.5Cr alloy with a tensile strength of 554 MPa and conductivity of 84% international annealed copper standard (IACS,0.49 S/cm) by ECAP + cold rolling + aging. 

Xu et al. [16] prepared ultra-fine grained Cu–Cr alloys by ECAP, which simultaneously improved strength, plasticity, and fatigue properties. Purcell et al. [17] found that after ECAP deformed eight passes and aging treatment, the tensile strength of the Cu–Cr–Zr alloy increased greatly from 189 MPa to 661 MPa, and the electrical conductivity kept no obvious variant. Tang et al. [18] revealed that after ECAP deformation at 450 °C, the tensile strength of the Cu–Cr–Zr alloy reached 580 MPa and the electrical conductivity reached 81% IACS (0.47 S/cm). Xia [19] et al. found that there was a faster and larger increase in resistivity of the peak-aged Cu–Cr–Zr–Mg–Si alloy during the deformation process. From previous studies, it can be believed that increase in the strength and conductivity is mainly attributed to the ultra-fined grains (UFG) resulted by ECAP and the nanoscale particles along the grain boundaries (GBs), and the proper heat treatment process can promote the distribution of the precipitated phase in the matrix at the grain boundaries [19,20]. Nowadays, most of the research on Cu [21,22] and Cu–Cr alloys [23,24,25] have focused on the microstructure and mechanical properties during the deformation process, while the effect of the toughening mechanism and the conductivity evolution during severe plastic deformation (SPD) and heat treatment is still unclear. Christiansen et al. [26] conducted rapid solidification of Cu–Cr (~32 at%Cr) alloy by splat quenching or laser melting techniques and a comparison with severe plastic deformation, the mixing phenomenon of Cu and Cr with different lattice types is discussed. However, there is a lack of systematic research on Cu–Cr–Si alloys. In this work, trace Si element was added to the Cu–Cr alloy, then ECAP deformation and the aging treatment were carried out. The mechanical properties and electrical conductivity of the alloy were tested, and the mechanism of microstructure and texture on the mechanical properties and electrical conductivity was analyzed.

## 2. Materials and Methods

Cu–1Cr–0.2Si (mass fraction, wt.%) alloy was prepared by high-purity electrolytic copper Cu (99.9%), Cu–1.2Cr (mass fraction, wt.%), and a Cu–0.5Si (mass fraction, wt.%) intermediate alloy. The ingot was homogenized at 900 °C for 5 h, and then extruded into a rod 30 mm in diameter. After multiple passes of cold rolling and drawing into a rod 12 mm in diameter, the alloy rod was then processed into Φ12 mm × 70 mm. ECAP deformation was conducted at room temperature using a die (the inner angle Φ = 90°, outer angle Ψ = 37°) up to eight passes through route Bc (where the sample is rotated 90° in the same direction between two consecutive passes) at the rate of 10 mm/s. The specimens were sampled along the extrusion direction and then aged at 350 °C for 1 to 8 h, respectively. All specimens were polished and corroded at room temperature for 10 seconds (corrosion ratio: FeCl_3_: 5 g, HCl: 50 ml, H_2_O: 100 mL). Microstructures were performed by a Quanta FEG-450 scanning electron microscope (FEI, Hillsboro, OR, USA) equipped with INCAEDS software. A D8ADVANCE x-ray diffractometer (Bruker AXS GmbH, Karlsruhe, Germany) was used to characterize the macroscopic orientation. Using a WDW-300D microcomputer (DaDi KeYu JiXie SheBei Co., Ltd, Beijing, China) controlled electronic universal testing machine to complete the tensile tests to measure the tensile strength with an error range of ±0.5%. The conductivity was tested by a Sigma2008B/C digital eddy current metal conductivity meter (Tianyan Instrument Co., Ltd., Xiamen, China) with an error range of 0.1%.

## 3. Results

### 3.1. Microstructure Evolution

**Figure 1:**
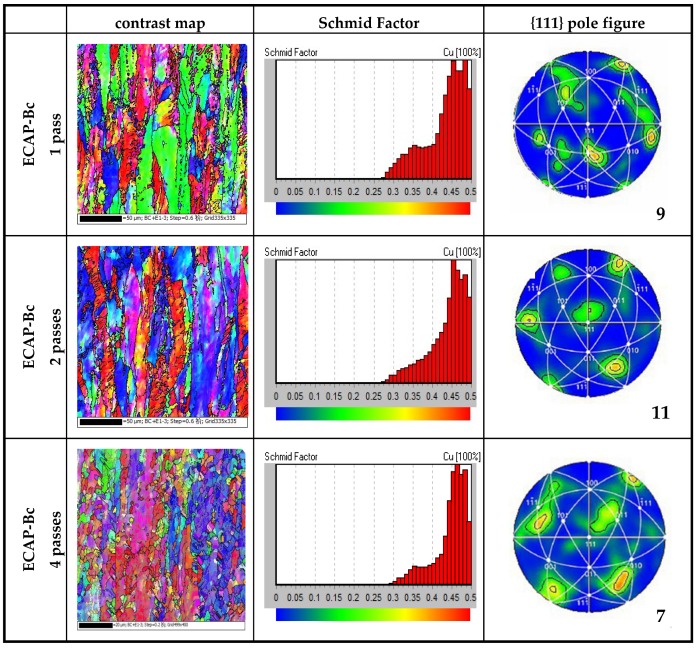

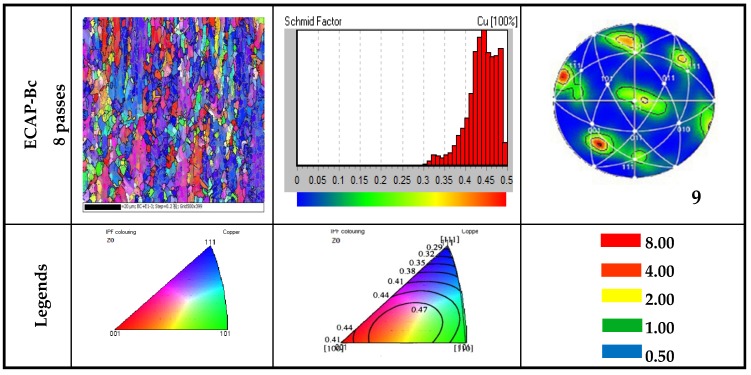
Contrast map, Schmid Factor, {111} pole figure for different passes of ECAP by route Bc.

Figure 1 shows the contrastmap, Schmid Factor, and the {111} pole figure for different passes of ECAP through route Bc. It can be seen from Figure 1 that after one pass of ECAP, the main orientation of the grains was {110} with high consistency; after two passes of deformation, a small equiaxed deformation structure appeared inside the grains, most of the crystals began to break, the GBs were extremely irregular, and the contrast between the grains increased significantly. This shows that the interaction between the GBs and the orientation rotation of the grains during the grain breaking process are conducive to improving the uniformity of the material structure and gradually making the orientation distribution uniform. After four passes of deformation, it can be seen that the GBs gradually decreased and the orientation tended to be consistent during the extrusion process. Accompanying the strain accumulated excessively, the preferred grain orientation changed from {110} to {111} and the grains were broken into 2 to 5μm. After eight passes of extrusion, the grain size was reduced to less than 2 μm. The corresponding Schmid factor showed that with an increase in the strain, the deformation in the range of 0.3–0.4 tended to be gentle, which indicates that the selected direction deflects toward the {111} crystal plane.

It can be seen from the {111} pole figures of Cu–1Cr–0.2Si alloy after different pass deformation and aging treatment at 350 °C for 4 h (most obvious), after one pass, the orientation segregated in the {110} plane and the pole density reached nine. After two passes of deformation and aging, the strongest orientation rotated about 45° counterclockwise around the extrusion direction, accompanied by strain increase, and the base texture deflected and the density fluctuated, which indicated that strong interactions occurred in the microstructure during deformation. When the strain was lower, the orientation tended to the same crystal plane, which was consistent with the previous structure. The occurrence of dispersion indicated that under high strain, the original coarse grains were gradually refined after being broken, the microstructure tended to be uniformed, and some subgrains were transformed into grains with high angle grain boundaries (HAGBs).

According to Figure 1 of one to two passes, the {111} crystal plane is the optimal orientation, the crystal plane rotates in an obvious orientation, and the rotation rules of the remaining orientations should be {101}→{001}→{111}. From our previous studies [27], the deformation band continuously reduced with the disappearance of grain boundaries (GBs) during the deformation process, and a large number of unsteady defects such as dislocations and vacancies appeared in the matrix, which gradually became stable during the subsequent annealing process. Deformation through the route Bc can promote the formation of cellular structure through dislocation entanglement under stress relaxation and gradually flatten the cell wall, which causes the alloy to easily recrystallize and lead to the transformation of the deformation bands [28,29].

### 3.2. Texture Evolution

**Figure 2:**
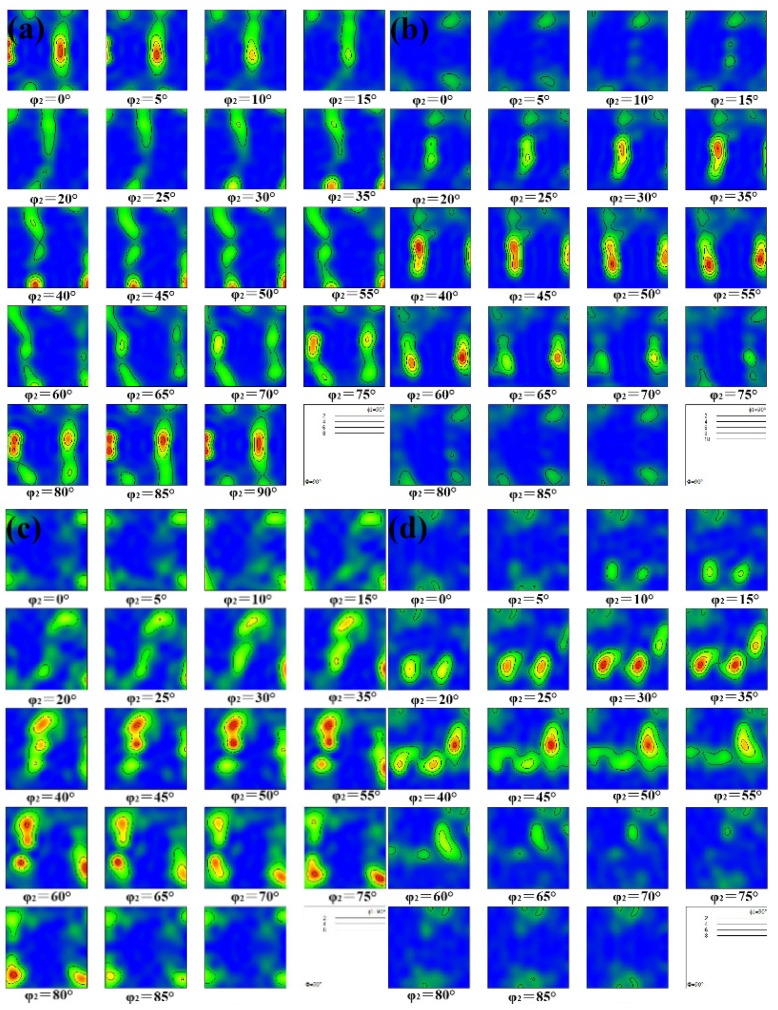
The ODF sections of the Cu–1Cr–0.2Si alloy after different passes deformation and aging at 350 °C for 4 h: (**a**) 1 pass; (**b**) 2 passes; (**c**) 4 passes; (**d**) 8 passes.

Figure 2 shows the orientation distribution function (ODF) of the Cu–1Cr–0.2Si alloy after different deformation passes and aging at 350 °C for 4 h (most obvious). It can be seen from the φ2 = 0° section that the ECAP deformation exhibited a strong {110}<001> texture after one pass, while the strength greatly decreased after four passes, and the weak {001}<110> rotating cubic texture formed after eight passes. The texture evolution was {001}<110>→{001}<100>→{001}<110>. It can be seen from the φ2 = 45° section that the texture transformation processes was {110}<001>→{111}<112>→{111}<110> and {111}<112>. The {hkl}<110> texture with a stable orientation gradually formed as the number of deformations increased. Previous studies have found that this texture is beneficial to improve the electrical conductivity of the materials [30]. At the same time, the ECAP deformation refines the microstructure and some crystal grains are broken into fine equiaxed crystals.

With the local recovery of the deformed structure, the aging treatment accelerates the precipitation process, which further causes the orientation to diverge and the texture strength to gradually decrease. For the different shear plane of adjacent passes by route Bc, the original texture had an effect on the texture that was produced by the first two passes of ECAP deformation [31]. After four deformation passes, multiple slip systems simultaneously started and the cross slip occurred, causing the texture to change continuously. Furthermore, the aging treatment caused the material to restore and recrystallize, further reducing the texture strength in some directions.

### 3.3. Misorientation and Dislocation Density

**Figure 3:**
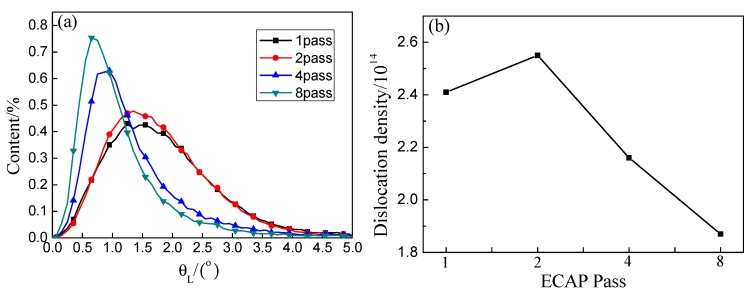
Distribution curve of local orientation difference and dislocation density under different deformation passes in aged Cu–1Cr–0.2Si alloy. (**a**) Local orientation difference distribution. (**b**) Dislocation density.

The local orientation difference reflected the orientation change in the plastic deformed zone. Although the dislocation density is not easy to measure directly, the relative size of dislocation density can be measured by the orientation change [32,33]. Therefore, the change in the orientation of the microstructure and the tendency of the dislocation density can be analyzed by the local orientation difference θ_L_. Figure 3 shows the curves of the orientation difference under different ECAP deformation passes and aging of the Cu–1Cr–0.2Si alloy. It can be seen from Figure 3a that under the different extrusion passes and the same aging state, the local orientation difference of the Cu–1Cr–0.2Si alloy had the characteristics of centralized distribution. One and two passes were mainly between 0° and 4°, while four passes and eight passes were mainly between 0° and 3°. With an increase in the deformation passes, the distribution orientation shifted to the left in turn, and the peak distribution of different passes appeared at 1.45°, 1.35°, 0.95°, and 0.65°. This shows that the misorientation of the microstructure becomes more and more concentrated as the number of deformations increased under the same aging conditions, this result is very consistent with Figure 1.

There is a quantitative conversion relationship between the local orientation difference θ_L_ and the dislocation density ρ at this point, which can be expressed by Kubin and Mortensen [34,35], as follows:

(1) $$\rho =2\theta_{L}/(\mu b)$$

where b is the mode of Burgers vector, which is 0.255; μ is the scanning unit and the length is 100 nm, and θ_L_ is the average value of the local orientation differences that can be obtained from the EBSD data.

The dislocation density of each deformation pass was calculated successively as ρ1 = 2.41 × 10^14^, ρ2 = 2.55 × 10^14^, ρ4 = 2.16 × 10^14^, and ρ8 = 1.87 × 10^14^. The change trend is shown in Figure 3b. It can be seen that the dislocation density of the alloy increased with the increase in the number of deformations [36], and the dislocation density of the aged alloy decreased with the increase in the strain. There may be a critical dislocation density value ρ0 in the deformed alloy under certain aging conditions. Once the value was exceeded, the alloy is prone to recovery and recrystallization under some aging condition. Dislocation movement intensified and dislocation rearrangement and annihilation speed accelerated during the recovery process. At the same time, dislocations were gradually absorbed by the subgrain boundaries as the aging time increased, thus making the dislocation density sharply decline.

### 3.4. Materials Characterization 

**Figure 4:**
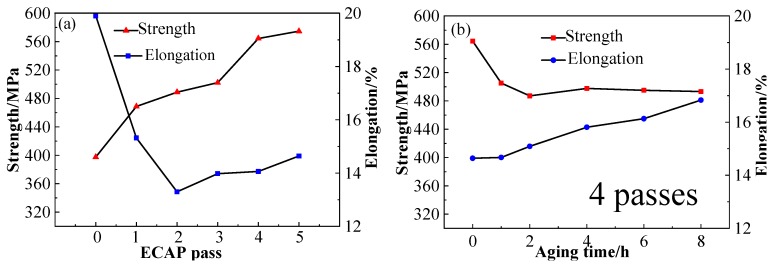
Mechanical properties of Cu–1Cr–0.2Si alloy. (**a**) ECAP deformation. (**b**) Aging treatment at 350 °C after four ECAP passes.

Figure 4a shows the results of the tensile strength and elongation of Cu–1Cr–0.2Si alloy during ECAP and aging treatment. From the curves in Figure 4a, it can be concluded that the tensile strength increased from 398 MPa to 575 MPa after five deformation passes, which was 45% higher than the initial strength. With an increasing number of deformation passes, the elongation declined at first, and then increased slightly, finally reaching 15% after five passes. Through analyses under low strain, deformation caused a large number of defects such as dislocations and subgrain boundaries in the alloy structure, and the dislocation density increased sharply, while high-density dislocations entangled and intersected with each other. Dislocation movement was affected by solute atoms, grain boundaries, and subgrain boundaries. Obstacles such as isostructure and precipitated phase increased the deformation resistance of the alloy and improved the strength and hardness of the material. In [37], in the late stage of deformation, the dislocations continued to multiply and annihilate, and the dislocation density gradually reached a dynamic equilibrium state, refined, and formed a fibrous structure along the extrusion direction, so that the strength can be maintained and slowly increased. These results are expected to rapidly increase the strength of the alloy, and make the alloy discontinuously distributed. The precipitated phase along the GBs further increased the strength of the material [38]. With the increase in strain, the microstructure of the alloy refined to the sub-micron or even nano-scale, and GBs as the source of dislocations, were conducive to the smooth progress of plastic deformation [39,40]. Meanwhile, the improvement of microstructure uniformity was conducive to elevate the materials’ plasticity. Figure 4b shows the evolution curves of tensile strength and elongation of the Cu–1Cr–0.2Si alloy after ECAP for four passes and aged at 350 °C. From the curves in Figure 4b can be concluded after four deformation passes and 4 h of aging, the tensile strength decreased to 498 MPa, which was 25% higher than the original state. Accompanying the UTS tended to be stable. The elongation of the alloy increased to 17% after eight hours of aging treatment.

**Figure 5:**
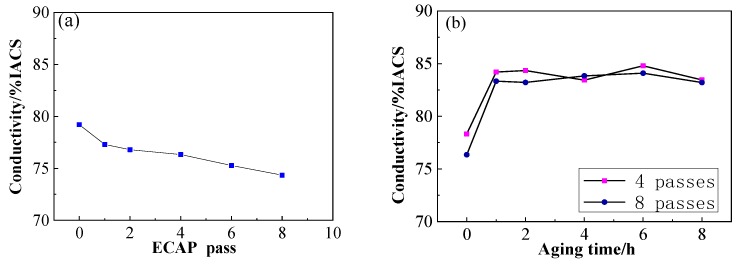
Electrical conductivity of Cu–1Cr–0.2Si alloy. (**a**) ECAP deformation of one to eight passes and (**b**) aging treatment at 350 °C for 8 h.

Figure 5a shows the evolution curves of the electrical conductivity of the Cu–1Cr–0.2Si alloy through the ECAP route Bc deformation and aging treatment. It can be seen that the electrical conductivity of the alloy slightly decreased from 79% to 74% IACS (0.43 S/cm) after eight passes with an increase in the deformation passes. Figure 5b shows the evolution curves of the alloy after ECAP deformations of four and eight passes and then aged at 350 °C for 1 to 8 h. The curves can be found that the conductivity rapidly rose to exceed 83% IACS (0.48 S/cm) after aging for 1 h, and then remained stable. This is obviously different from the test results from Xia et al. [19]. 

## 4. Discussion

### 4.1. Mechanical Properties

During ECAP deformation through the route Bc, the microstructure of the Cu–1Cr–0.2Si alloy was gradually refined into a fibrous structure, and then distributed along the extrusion direction. The high-density dislocations introduced by the deformation and lots of unstable subgrain boundaries and GBs caused by deformation lead to a continuous increase in lattice distortion energy, leading to an increase in the resistance of the free electrons to move, as shown in Figure 5a, resulting in a slight decrease in the electrical conductivity. The effect of temperature-rise led to the precipitation of a large number of enhanced phases near the GBs and deformation zones, pinning dislocations and increased lattice distortion, and some irregular Cr phases further increased the lattice mismatch between the matrix and the heterogeneous interface. The precipitated phase caused by ECAP deformation was mainly distributed near the GBs and the deformation bands hindered the movement of dislocations, which is conducive to increasing the strength and decreasing the elongation continuously. In the early stages of aging treatment, dislocations annihilated, and work hardening was gradually eliminated. The continuous increase in the precipitated phase led to the increase of dislocation movement resistance, thereby further maintaining the strength of the alloy and partially recovering the plasticity. With an increase in aging time, the density of defects (such as dislocations) decreased, which reduced the effect of deformation strengthening, leading to a decrease in material strength. Precipitation strengthening begins to play an important role in affecting the strength of the alloy being tested. Studies [41] have shown that the Cr-rich face-centered cubic phase of the Cu–Cr alloy precipitated during the aging process, and then formed the Cr precipitated phase with a body-centered cubic structure, which maintained a coherent or semi-coherent relationship with the copper matrix. In the later stage of aging, the unbalanced growth of the precipitated phase destroyed the coherent relationship of the matrix and precipitated phase, increased the distortion energy, and was not conducive to improve the mechanical properties. 

### 4.2. Electrical Conductivity

Previous studies have found that deformation and aging treatment directly affect the electrical conductivity of materials [42]. In the early stage of aging, defects such as vacancies and dislocations began to annihilate, and the unsteady structure such as subgrain boundaries gradually became stable while the solid solution atoms began to quickly nucleate. This shows that this process reduces the scattering of free electrons, which makes the conductivity rise sharply. With the prolonging of aging time, the precipitation phase grows and maintains a coherent relationship with the matrix, resulting in a fluctuating electrical conductivity. In the later stage of the aging process, the precipitation phase with a large size gradually destroys the coherence relationship with the matrix, resulting in a decrease in the distortion energy. During the recovery process, as the density of dislocation in the crystal continues to decrease, it is beneficial to maintain a higher conductivity of the material. From the changing trends, the curves in Figure 4 and Figure 5 can be found by optimizing the deformation and heat treatment process, so the strength, elongation, and conductivity of the alloy are expected to further improve. This work will be carried out further in the future.

## 5. Conclusions

(1) A high strength, high electrical conductivity, and high elongation Cu–1Cr–0.2Si alloy was fabricated by ECAP through route Bc + aging treatment (the elongation reached 17%, strength about 500 MPa, the electrical conductivity reached 83% IACS (0.48 S/cm)), and the strength of the alloy is expected to be further improve. 

(2) With an increase in the ECAP deformation passes, the tensile strength of the Cu–1Cr–0.2Si alloy was continuously increased. After four deformation passes, it reached 564 MPa, which was 42% higher than the undeformed material.

(3) Aging treatment can promote the dispersion of Cr phases in the matrix, and greatly increase the conductivity of the Cu–1Cr–0.2Si alloy. With an increase in aging time, the quantity of the phases increased gradually and was uniformly dispersed in the matrix.

(4) The strong {111}<110> and {111}<112> deformed textures and weak {001}<100> recrystallized textures were formed after eight passes of deformation. ECAP deformation and aging treatment can greatly increase the strength and conductivity of the material. The stable {111}<110> texture after ECAP and the lower dislocation density after aging treatment are the main reasons for the high conductivity of the material.
